# Estimation of the effects of heavy Asian dust on respiratory function by definition type

**DOI:** 10.1186/s41021-017-0085-9

**Published:** 2017-11-01

**Authors:** Jun Kurai, Masanari Watanabe, Hisashi Noma, Kyoko Iwata, Jumpei Taniguchi, Hiroyuki Sano, Yuji Tohda, Eiji Shimizu

**Affiliations:** 1 Department of Respiratory Medicine and Rheumatology, Faculty of Medicine, Tottori University, 36-1 Nishi-cho, Yonago, Tottori 683-8504 Japan; 2 Department of Data Science, The Institute of Statistical Mathematics, 10-3 Midori-cho, Tachikawa, Tokyo, 190-8562 Japan; 3 Mio Fertility Clinic, Reproductive Centre, Tottori, Japan; 4 Department of Respiratory Medicine and Allergology, Kinki University Faculty of Medicine, 377-2 Ohnohigashi, Osakasayama, 589-0014 Japan

**Keywords:** Asian dust, Asthma, Peak expiratory flow, Respiratory function, School children

## Abstract

**Background:**

The adverse effects of Asian dust (AD) on health have been demonstrated in earlier studies, but there is no standardized definition for heavy–AD. This study aimed to examine which definition of heavy–AD has the most adverse effect on respiratory function.

**Methods:**

One–hundred–and–thirty–seven adults with asthma, and 384 school children self-measured their morning peak expiratory flow (PEF). The four definitions of heavy–AD are: (1) the definition provided by the Japan Meteorological Agency (JMA), (2) daily median AD particle level ≥ 0.07 km^−1^, obtained through light detection and ranging (LIDAR) (3) hourly AD particle level ≥ 0.1 km^−1^, and (4) hourly level ≥ 0.07 km^−1^. Linear mixed models were used to estimate the effects of heavy–AD, by definition type, on daily PEF values.

**Results:**

In adults with asthma, as per the JMA’s definition, significantly reduced PEF were observed on heavy–AD days (lag 0), lag 0–1, and lag 0–3. In school children, after a heavy–AD event, as defined by the JMA, PEF significantly decreased on lag 0–1, lag 0–2, and lag 0–3. However, as per the other definitions, there was no significant decrease in the PEF in the adults and children.

**Conclusion:**

The associations between heavy–AD and respiratory function differed between these definitions.

## Background

Asian dust (AD) originates in the deserts of Mongolia, northern China, and Kazakhstan, and is the second largest type of sand dust emission in the world [1]. Therefore, based on the prevailing winds, AD disperses eastward and passes over China, North and South Korea, and the eastern parts of Russia, and Japan. Over the past few decades, AD has become a serious problem due to the rapid increase in the contents of industrial pollutants, brought on by the emission from expanding industries and the increasing number of cars in East Asia [2–7]. A number of studies have demonstrated that AD aggravates mortality and increases the requirement for emergency treatment and hospitalization for cardiovascular disease and pulmonary disease [8–11]. Similarly, AD was associated with an increased risk of hospitalization and exacerbation, in the case of asthma [12–15]. AD can also decrease respiratory function in children with and without asthma [16, 17].

Depolarization light detection and ranging (LIDAR) provides air quality measurements by two wavelengths that are simultaneously applied within <1 km above the ground [18, 19]. The LIDAR system detects sand dust particles and aerosolized air pollutants, using the extinction coefficient. Therefore, LIDAR can measure the levels of AD in real time. According to LIDAR data, AD particles are carried to Japan on most days from March to May [15, 18, 19].

Heavy–AD days were defined in almost all the studies that estimated the association between AD and health disorders. Strictly speaking, this means that almost all these studies focused on the effects of heavy–AD on health. However, the definition of heavy–AD differs among countries [20]. In Japan, there is currently no standardized definition for heavy-AD. In recent times, many studies from Japan have been using LIDAR data to define heavy–AD [13, 21, 22]. However, LIDAR systems lack defined criteria for heavy–AD. Therefore, despite using LIDAR data to determine heavy-AD, this definition varies for each study.

Another study conducted by our team found that heavy–AD has adverse effects on the respiratory function of school children and adults with asthma [14, 15, 23, 24]. However, these studies used a single definition of heavy–AD to estimate the association between heavy–AD and respiratory function. This study aimed to examine whether these differences in the definitions of heavy–AD lead to consequent differences in the effects of heavy–AD on respiratory function, by further analyzing data obtained from our earlier studies (2013), in which we investigated the effects of heavy–AD exposure on respiratory function in school children and adults with asthma [23, 24].

## Methods

### Study design

**Fig. 1:**
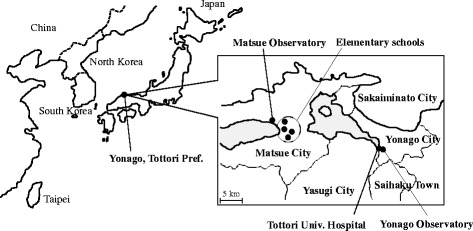
Location of the Tottori University Hospital, elementary schools, residential areas, and observatories

To estimate the effects of AD on respiratory function in school children and adults with asthma, panel studies were conducted, in which the daily peak expiratory flow (PEF) values were measured, from March to May 2013. One hundred and thirty-seven adults with asthma, aged >18 years, and 384 school children aged 9 and 10 years were enrolled. The adults resided in Yonago, Matsue, Sakaiminato, Yasugi, or Saihaku, all of which are located within 25 km of Tottori University Hospital in Yonago, Tottori, western Japan as shown in Fig. 1 [23]. Based on the criteria of the Global Initiative for Asthma (GINA) [25], these participants were diagnosed as having asthma if they presented with a history of intermittent wheezing and exhibited airway hyperresponsiveness to methacholine, or exhibited reversible airflow limitations (12% and 200–mL variability in the forced expiratory volume in 1 s [FEV_1_]). The study was approved by the institutional ethics committee (Ethics Committee of Tottori University, approval no. 1656), and all the patients provided written informed consent.

School children from four of a total of 35 elementary schools in Matsue, Shimane, were enrolled, in 2013 (Figure 1) [24]. The four elementary schools were within 10 km of each other and all the participants lived within a radius of 1 km from the schools. Data on the participants’ age, gender, height, and weight, as well as the presence of asthma, allergic rhinitis, allergic conjunctivitis, atopic dermatitis, and food allergies, were recorded in March 2013. The study was approved by the institutional ethics committee (Ethics Committee of Tottori University, Approval Number 1764). The children and their parents were informed of the study by teachers, and the school children and their parents provided written informed consent for participation.

### Recording daily PEF values

The PEF values were measured using a peak flow meter (Mini-Wright, Harlow, England, American Thoracic Society scale). Every day, from February to May 2013, all the participants recorded their morning PEF values. February was assigned as the trial period. PEF values were measured three times in the morning, and participants recorded the best of the three obtained values. Participants with asthma measured their PEF values before taking their medication.

### Measurement of air pollutants and meteorological variables

Data on the concentrations of nitrogen dioxide (NO_2_), ozone, particulate matter smaller than 2.5 μm (PM_2.5_), sulfur dioxide (SO_2_), and suspended particulate matter (SPM) are monitored at many locations in Japan, by the Japanese Ministry of the Environment. For the current analysis, data on the concentrations of SPM, SO_2_, and NO_2_ were obtained from the Yonago and Matsue observatories. The Matsue observatory, under the Japanese Ministry of the Environment, has a LIDAR system and so, LIDAR data were obtained from there. Values that were measured at 120–150 m above the ground, which is the minimum altitude required by LIDAR systems to measure AD particles, were used. Data on daily temperature, humidity, and atmospheric pressure in Yonago and Matsue were obtained from the Japan Meteorological Agency (JMA).

### Definition of heavy–AD

At present, in Japan, there is no standardized definition for heavy–AD. The JMA, with reference to data obtained from meteorological satellites (http://www.jma.go.jp/jp/kosa/), defines a heavy–AD day as a day on which visibility is reduced to <10 km, due to sand dust arising from the deserts of East Asia. LIDAR systems measure the levels of AD particles at 15-min intervals [18, 19]. Usually, the daily levels of the AD particles were on the median value of 96 measurements collected over a 24 h period, but they were omitted when the number of available measurements fell below 50% of the total number of measurements. Ueda et al. reported that a cut-off level of 0.07 km^−1^ in the median value within 24 h was associated with an increase in the number of emergency ambulance dispatches [26]. The hourly value is presented on the Japanese Ministry of the Environment’s website (http://soramame.taiki.go.jp/dss/kosa/). An hourly value of 0.1 km^−1^, which corresponds to a visibility range of 10 km [27] and 0.1 mg/m^3^ of sand dust particles [19], is the lowest detectable value in real time. In the present study, in order to examine whether differences in the definition of heavy-AD lead to different health outcomes, four cut-off levels of heavy-AD days were defined: (1) the JMA’s aforementioned definition (2) 24–h median AD particle levels greater than 0.07 km^−1^, (3) hourly AD particle levels greater than 0.1 km^−1^, and (4) hourly AD particle levels greater than 0.07 km^−1^. As per the JMA’s definition, there were five heavy–AD days– March 8–10, and March 19 and 20, between March and May 2013, in both Matsue and Yonago. There were 5 days during the study period– March 7, March 9, March 20, and May 30 and 31– in which the 24-h median AD particle levels were more than 0.07 km^−1^. There were 8 days– March 7–10, March 17, March 20, April 6, and April 30, which met the criteria (hourly AD particle levels greater than 0.1 km^−1^). There were 20 heavy–AD days– March 7–10, March 16-20, April 5, 6, 9, 10, 16, 29, and 30, and May 30 and 31– on which the hourly AD particle levels were greater than 0.07 km^−1^.

### Statistical analysis

For the analyses of longitudinal repeated measurement data, linear mixed models were used to estimate the effects of heavy–AD on the daily PEF values of the adults with asthma as well as the school children [28, 29]. Similarly, the relationship between PEF values and the daily median levels of AD particles, according to LIDAR data, were estimated using the mixed effects models. In the analyses of the adults with asthma, the individual characteristics were included as adjustment factors in the regression models; age, sex, smoking status, the presence of allergic rhinitis and atopic disposition, treatment step, and respiratory function. In addition, the meteorological data (temperature, humidity, atmospheric pressure) of Yonago were included as adjustment factors. Moreover, in the analyses of the data on school children, the potential confounding factors were also adjusted in the regression models: age, sex, height, weight, the presence of asthma, allergic rhinitis, allergic conjunctivitis, atopic dermatitis, food allergies, and the meteorological variables measured in Matsue. After that, we involved random intercepts, which adequately assess intra-individual correlations among the repeated measurements.

The effects of heavy–AD days on PEF values were compared to those of non–heavy AD days. To evaluate potential persistent effects of heavy–AD on respiratory function, the lagged cumulative effects were analyzed for lag day 0 (the day of the heavy–AD event), lag day 0 to day 1 (defined as lag 0–1), lag day 0 to day 2 (lag 0–2), and lag day 0 to day 3 (lag 0–3). Estimates were provided as the regression coefficient of PEF values per interquartile range (IQR) change in AD particle levels, with 95% confidence intervals (CIs). The multiple imputation method was used to treat missing data that adequately addressed the uncertainty of the imputed values, based on multiple generated prediction values for missing data [30]. R version 3.3.2 (R Foundation for Statistical Computing, Vienna, Austria) was used for the analyses using the linear mixed models. The t–test was performed to compare the levels of air pollutants between heavy–AD days and non–heavy AD days in each heavy-AD definition. One-way analysis of variance (ANOVA) was used to determine whether there were any statistically significant differences in the levels of NO_2_, ozone, SO_2_, PM_2.5_, and SPM among the four definitions of heavy–AD days. The t–test and ANOVA were performed with SPSS software (Japanese version 22.0 for Windows; SPSS Japan Inc., Tokyo, Japan). All the *P* values were two-sided, and the significance level was set at <0.05.

## Results

### Participant characteristics

The characteristics of the adults with asthma are shown in Table 1. Based on Japan’s Asthma Prevention and Management Guideline, 2012, the treatment step which corresponded to the participants’ asthma control levels was used in March 2013 [31]. Table 2 presents the characteristics of the school children.

**Table 1:** Characteristics of the 137 adults with asthma

Male/female	58 (42.3)/79 (57.3)
Age (years)	63.5 ± 15.4
Never smoked	92 (67.1)
Former smoker	38 (27.7)
Current smoker	7 (5.2)
Respiratory function	
FVC (L)	2.94 ± 0.70
FEV_1_ (L)	2.09 ± 0.60
%FEV_1_ (%)	100.4 ± 24.7
Atopic disposition	74 (54.0)
Allergic rhinitis and/or chronic sinusitis	60 (43.8)
Treatment step	
Step 1	1 (0.7)
Step 2	14 (10.2)
Step 3	29 (21.2)
Step 4	87 (63.5)
Step 5	6 (4.4)

Data are shown as the frequency (percentage) of adults with asthma or the mean ± standard deviation. FEV_1_: forced expiratory volume in 1 s; %FEV_1_: percentage of predicted FEV_1_; FVC: forced vital capacity

**Table 2:** Characteristics of the 384 school children

Boys/girls	194 (50.5)/190 (49.5)
Height (cm)	137.7 ± 7.0
Boys/girls (cm)	136.9 ± 6.3/138.5 ± 7.7
Weight (kg)	32.4 ± 6.6
Boys/girls (kg)	32.3 ± 6.8/32.6 ± 6.4
Allergic disease	
Asthma	45 (11.7)
Allergic rhinitis	74 (19.3)
Allergic conjunctivitis	15 (3.9)
Atopic dermatitis	36 (9.4)
Food allergy	20 (5.2)

Data are shown as the frequency (percentage) of school children or the mean ± standard deviation

### Levels of air pollutants

The levels of gaseous pollutants were classified into those on heavy–AD and non-heavy AD days, according to each heavy-AD definition, as shown in Table 3, using the data from Matsue. There was a significant difference in the SO_2_ levels, between heavy–AD and non–heavy–AD days, as determined by the JMA definition. When a heavy–AD day was defined as hourly AD particle levels greater than 0.07 km^−1^ (using LIDAR), there were significant differences in the levels of NO_2_, ozone, and SO_2_ between heavy–AD and non–heavy AD days. The levels of NO_2_, ozone, and SO_2_ on heavy–AD days, as defined by daily median and hourly levels greater than 0.07 km^−1^, did not significantly differ from those on non–heavy AD days. There was no significant difference in the levels of NO_2_, ozone, and SO_2_, between the four definitions of heavy–AD days. Table 4 presents the levels of particulate air pollution. In all four definitions of heavy–AD days, the levels of SPM and PM_2.5_, were significantly higher than those on non–heavy AD days. There was no significant difference in the levels of SPM and PM_2.5_ among the four definitions of heavy–AD days.

**Table 3:** Levels of gaseous pollutants on heavy–AD and non–heavy AD days, by definition type

Type of day (number of days)	NO_2_ (ppb)		Ozone (ppb)		SO_2_ (ppb)	
Heavy–AD days, as per the definition by the JMA (*n* = 5)	3.5	± 1.3	57.6	± 10.9	2.0	± 1.0
Non–heavy AD days, as per the definition by the JMA (*n* = 87)	2.8	± 1.2	50.1	± 8.4	1.0	± 0.7
Heavy–AD days defined by daily median level ≥ 0.07 km^−1^ (detected by LIDAR) (n = 5)	3.7	± 1.3	52.2	± 10.4	1.3	± 0.9
Non–heavy AD days, defined by daily median level ≥ 0.07 km^−1^ (detected by LIDAR) (*n* = 87)	2.7	± 1.1	50.4	± 8.6	1.0	± 0.8
Heavy–AD days, defined by hourly level ≥ 0.1 km^−1^ (detected by LIDAR) (n = 8)	3.3	± 1.2	52.9	± 10.5	1.4	± 0.9
Non–heavy AD days, defined by hourly level ≥ 0.1 km^−1^ (detected by LIDAR) (*n* = 84)	2.8	± 1.2	50.3	± 8.5	1.0	± 0.8
Heavy–AD days, defined by hourly level ≥ 0.07 km^−1^ (detected by LIDAR) (*n* = 20)	3.0	± 1.0	54.3	± 9.2	1.4	± 0.8
Non–heavy AD days, defined by hourly level ≥ 0.07 km^−1^ (detected by LIDAR) (*n* = 72)	2.7	± 1.2	49.5	± 8.3	0.9	± 0.8

*AD* Asian dust, *JMA* Japan Meteorological Agency, *LIDAR* light detection and ranging, *NO*_*2*_ nitrogen dioxide, *SO*_*2*_ sulfur dioxide

**Table 4:** Particulate air pollution levels on heavy–AD and non–heavy AD days, by definition type

Type of day (number of days)	SPM (μg/m^3^)		PM2.5 (μg/m^3^)	
Heavy–AD days, as per the definition by the JMA (n = 5)	39.6	± 15.1	37.5	± 12.1
Non–heavy AD days, as per the definition by the JMA (n = 87)	17.9	± 8.9	17.6	± 7.3
Heavy–AD days, defined by daily median level ≥ 0.07 km^−1^ (detected by LIDAR) (n = 5)	35.0	± 16.7	31.3	± 15.6
Non–heavy AD days, defined by daily median level ≥ 0.07 km^−1^ (detected by LIDAR) (*n* = 87)	18.1	± 9.3	18.0	± 7.9
Heavy–AD days, defined by hourly level ≥ 0.1 km^−1^ (detected by LIDAR) (n = 8)	31.8	± 17.0	29.5	± 14.5
Non–heavy AD days, defined by hourly level ≥ 0.1 km^−1^ (detected by LIDAR) (n = 84)	17.8	± 8.8	17.7	± 7.5
Heavy–AD days defined by hourly level ≥ 0.07 km^−1^ (detected by LIDAR) (n = 20)	28.1	± 12.6	27.1	± 10.8
Non–heavy AD days, defined by hourly level ≥ 0.07 km^−1^ (detected by LIDAR) (n = 72)	16.6	± 8.2	16.6	± 6.9

*AD* Asian dust, *JMA* Japan Meteorological Agency, *LIDAR* light detection and ranging, *PM*_*2.5*_ particulate matter smaller than 2.5 μm, *SPM* suspended particulate matter

### PEF changes

The effect of heavy–AD days on PEF values, in comparison to that of non–heavy AD days, in the adults, is shown in Table 5. As per the JMA’s definition, the PEF values were significantly lower on heavy–AD days than on non–heavy AD days. To facilitate the evaluation of the effects of heavy-AD on PEF values, the changes are shown on lag 0, lag 0–1, and lag 0–3. The lowest PEF values were observed on lag 0. Although lag 0–2 was an exception, the values gradually increased with time. No significant change was observed in the case of the other three heavy–AD definitions. Table 6 presents the relationship between changes in the PEF values and heavy–AD days in the school children. As per the JMA definition, there were significant decreases in PEF values 0 to 3 days after the heavy–AD event, and the values gradually increased with time. In contrast, heavy–AD days, as defined by daily median levels ≥0.07 km^−1^ (using LIDAR) were associated with significant increases in the PEF values, compared to non–heavy AD days. There were no significant effect modifications between the other two heavy–AD definitions. Table 7 shows the results of the changes in PEF values, per IQR increase in the levels of AD particles. No significant associations were observed in both the adults and school children.

**Table 5:** The effect of heavy–AD days on PEF values compared to that of non–heavy AD days, in adults with asthma

	Type of heavy–AD definition			
	JMA definition	Daily median level ≥ 0.07 km^−1^ (using LIDAR)	Hourly level ≥ 0.1 km^−1^ (using LIDAR)	Hourly level ≥ 0.07 km^−1^ (using LIDAR)
Lag time (days)	Change in PEF (L/min) [95% CI]	Change in PEF (L/min) [95% CI]	Change in PEF (L/min) [95% CI]	Change in PEF (L/min) [95% CI]
Lag 0	−1.76^a^ [−3.30, −0.21]	0.85 [−0.86, 2.57]	−0.18 [−1.61, 1.25]	0.38 [−0.60, 1.36]
Lag 0–1	−1.54^a^ [−2.84, −0.25]	−0.09 [−1.48, 1.29]	−0.21 [−1.42, 0.99]	0.35 [−0.53, 1.24]
Lag 0–2	−1.05 [−2.21, 0.11]	0.14 [−1.11, 1.38]	0.11 [−0.95, 1.17]	0.40 [−0.44, 1.23]
Lag 0–3	−1.09^a^ [−2.18, −0.01]	−0.04 [−1.19, 1.11]	0.18 [−0.82, 1.17]	0.11 [−0.70, 0.93]

*AD* Asian dust, *CI* confidence interval, *JMA* Japan Meteorological Agency, *LIDAR* Light detection and ranging, *PEF* peak expiratory flow

^a^ < 0.05 versus non-heavy AD days

**Table 6:** The effect of heavy–AD days on PEF values compared to that of non–heavy AD days, in school children

	Type of heavy–AD definition			
	JMA definition	Daily median level ≥ 0.07 km^−1^ (using LIDAR)	Hourly level of LIDAR ≥0.1 km^−1^ (using LIDAR)	Hourly level of LIDAR ≥0.07 km^−1^ (using LIDAR)
Lag time (days)	Change in PEF (L/min) [95% CI]	Change in PEF (L/min) [95% CI]	Change in PEF (L/min) [95% CI]	Change in PEF (L/min) [95% CI]
Lag 0	−2.33 [−5.09, 0.44]	3.53^a^ [0.85, 6.20]	−1.85 [−4.56, 0.86]	0.81 [−0.87, 2.50]
Lag 0–1	−2.72^a^ [−4.80, −0.53]	2.47^a^ [0.32, 4.63]	−1.63 [−3.58, 0.33]	0.95 [−0.48, 2.37]
Lag 0–2	−2.26^a^ [−3.98, −0.53]	1.69 [−0.15, 3.54]	−0.98 [−2.64, 0.67]	1.37 [0.01, 2.73]
Lag 0–3	−3.04^a^ [−4.68, −1.40]	1.25 [−0.50, 3.00]	−1.90^a^ [−3.50, −0.30]	1.11 [−0.23, 2.45]

*AD* Asian dust, *CI* confidence interval, *JMA* Japan Meteorological Agency, *LIDAR* light detection and ranging, *PEF* peak expiratory flow

^a^ < 0.05 versus non-heavy-AD days

**Table 7:** Associations between PEF and AD particles, based on LIDAR data

Subject	IQR (km^−1^)	Change in PEF (L/min)	95% CI	*P* value
Adults with asthma	0.02	0.01	−0.62, 0.11	NS
School children	0.02	0.27	−0.38, 0.92	NS

*AD* Asian dust, *CI* confidence interval, *IQR* interquartile range, *LIDAR* light detection and ranging, *NS* not significant, *PEF* peak expiratory flow

## Discussion

Because there is currently no standardized definition for heavy–AD, the present study determined four heavy–AD criteria, according to previously conducted studies, to examine whether differences in the definitions of heavy–AD corresponded to differences in the effects of heavy–AD on respiratory function. First, the present study chose the heavy–AD criteria, as determined by the JMA as well as hourly AD particle levels greater than 0.1 km^−1^, because data on the presence of heavy–AD in real time can be obtained through the corresponding website and thereby, the adverse effects of heavy-AD can be avoided. Other heavy–AD criteria, based on 24–h median AD particle levels greater than 0.07 km^−1^, were adopted because this definition was significantly associated with an increase in the number of emergency ambulance dispatches [26]. The present study also investigated heavy–AD defined by hourly AD particle levels greater than 0.07 km^−1^ and respiratory function. Heavy–AD, as defined by the JMA, had a significant and negative association with PEF values, in both the adults with asthma and school children. On the contrary, heavy–AD, as defined by a daily median level ≥ 0.07 km^−1^ (using LIDAR) increased the PEF values in these children. These results suggest that the effects of heavy–AD on respiratory function vary with the definition of heavy–AD.

There was no significant difference in the levels of NO_2_, ozone, and SO_2_ among the four definitions of heavy–AD. It was suggested that these meteorological factors and air pollutants did not influence the differences in the effects of heavy–AD on respiratory function, irrespective of the definition. Based on the JMA definition, Onishi et al. classified heavy–AD into three types: Type 1 with higher counts of air pollutant particles than sand dust particles; Type 2 with higher counts of sand dust particles than air pollutant particles; and Type 3 with very low counts of air pollutant particles [5]. Onishi et al. also reported that anthropogenic metals were important constructs of Type 1 heavy–AD. Based on data obtained from meteorological satellites, the JMA judged heavy–AD as that which causes visibility of less than 10 km. However, this method did not take into consideration the fact that air pollutant particles, moisture and steam also affect the visual range. Unlike the definition by the JMA, the other three definitions of heavy–AD factor in sand dust particle levels, detected by LIDAR. Therefore, the JMA’s definition of heavy-AD may include more air pollutant particles than the other three, according to LIDAR data. Air pollutants particles, in addition to sand dust particles, may also affect the decrease in the PEF values induced by heavy–AD– as defined by the JMA– because it was found that there was no relationship between daily levels of AD particles and PEF values in both adults with asthma and school children.

In school children, heavy–AD as defined by a daily median level ≥ 0.07 km^−1^ significantly increased PEF values. The reason for this is unclear. Although there was no relationship between PEF values and heavy–AD, as defined by daily median and hourly levels ≥0.07 km^−1^, the changes in the PEF values (lag 0) were higher than on non–heavy AD days, in both the adults with asthma and school children. Heavy–AD, based on daily median and hourly levels ≥0.07 km^−1^ occurred in May, during the study period, but there were no occurrences of heavy-AD in May, as per the JMA’s definition as well as the hourly levels ≥0.1 km^−1^. Weinmayr et al. revealed in their systematic review and meta–analysis that the effects of PM ≤ 10 μm (PM_10_) on respiratory health differed between seasons [32]. Thus, seasonal differences may have affected the results of the present study.

Other studies have defined heavy–AD as a daily median level ≥ 0.1 km^−1^ [13, 33]. These studies found that heavy–AD was associated with an increased risk of hospitalization in children with asthma [13] and emergency ambulance dispatches due to illnesses such as cardiovascular stress [33]. During this study period, there was no day on which the daily median level was >0.1 km^−1^. Therefore, the present study was unable to estimate the effects of heavy–AD, as defined by daily median level > 0.1 km^−1^, on respiratory function.

Based on the JMA criteria, there was a reduction in the difference of PEF values from 1.09 L/min to 3.04 L/min between heavy-AD days and non-heavy days. The difference may be small. Therefore, although there was a statistically significant difference in the PEF values between heavy–AD days and non–heavy AD days using the JMA criteria, this difference might be attributable to the error in the statistical analysis due to relative variations during PEF examinations and the interactive effects of the combinations. However, based on the JMA’s definition, the PEF values were significantly lower on heavy–AD days than on non-heavy AD days in the two different populations. Our previous studies showed that the frequency of asthma exacerbation from exposure to heavy–AD, based on the JMA’s definition in adult patients ranged from 11% to 22% [14, 34]. There was no effect of heavy-AD on the respiratory symptoms and respiratory function in most of the adult patients with asthma. There may be a susceptibility factor for AD. Compared to school children without asthma, there was a strong tendency toward a decrease in PEF values after exposure to heavy-AD in those with asthma [24]. In the present study, the minority of school children had asthma (11.7%). This could have accounted for the small reduction in the PEF values during heavy-AD.

There are several limitations to the present study. First, the interpretation was limited by the low incidence of heavy–AD days during the study period, as per the JMA’s definition, as well as the daily median level ≥ 0.07 km^−1^. Second, the levels of AD were measured at 120–150 m above the ground, which is the minimum altitude required by LIDAR systems. The LIDAR data may not completely correspond to the levels of AD particles near the ground. In contrast, the levels of SPM and PM_2.5_ were measured near the ground. The levels of SPM and PM_2.5_, on heavy–AD days, as per all four definitions, were significantly higher than on non–heavy AD days. Therefore, it was acceptable to use LIDAR data to estimate the effect of AD on health. Third, the present study was unable to measure individual exposure levels to AD particles and air pollutants. However, compared to adult patients with asthma, the differences in the individual exposure levels to AD particles and air pollutants, among school children, may be small because they took part in group activities.

## Conclusion

The effects of heavy–AD on respiratory function differed by heavy–AD definition. Heavy–AD, as defined by the JMA, significantly decreased respiratory function in both adults with asthma and school children. To reduce the adverse effects of AD on respiratory health, further studies are needed to determine a more suitable heavy–AD definition.

## Abbreviations

AD: Asian dust
FEV_1_: Forced expiratory volume in 1 s
GINA: Global Initiative for Asthma
JMA: Japan Meteorological Agency
LIDAR: Light detection and ranging
NO_2_: Nitrogen dioxide
PEF: Peak expiratory flow
PM: Particulate matter
PM_2.5_: Particulate matter smaller than 2.5 μm
SO_2_: Sulfur dioxide
SPM: Suspended particulate matter
